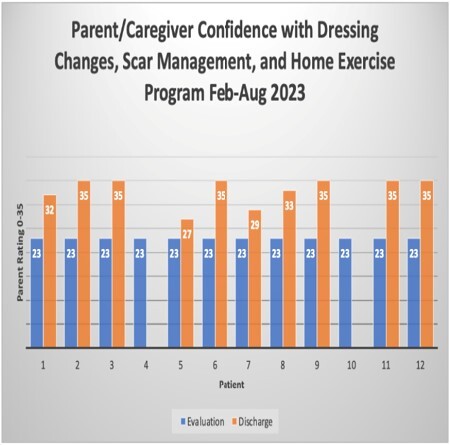# 751 Assessing Functional Outcomes in Pediatric Burn Acute Rehabilitation

**DOI:** 10.1093/jbcr/irae036.293

**Published:** 2024-04-17

**Authors:** Katie Coakley, AnnMarie Berry, Allison Davis

**Affiliations:** Loyola University Medical Center, Chicago, IL; Loyola University Medical Center, Maywood, IL; Loyola University Medical Center, Chicago, IL; Loyola University Medical Center, Maywood, IL; Loyola University Medical Center, Chicago, IL; Loyola University Medical Center, Maywood, IL

## Abstract

**Introduction:**

Pediatric burn survivors experience difficulty returning to daily activities. Functional outcome measures are needed for therapists to use in acute care. At one burn center the Center for Appearance Research (CARe) Burn Scales Parent Quality of Life Form and Boston Activity Measure for Post Acute Care (AM-PAC) were used with the pediatric burn population.

**Methods:**

Therapists used the AM-PAC with children 4-17. Therapists scored the child’s functional abilities at evaluation and discharge. The child’s ability to perform self-care was scored using the Daily Activity form and functional mobility was scored using the Basic Mobility form. Scores range from 6-24. Higher scores indicate greater independence. The AM-PAC was recently validated for use with the pediatric population, ages 4 and up. The project’s inclusion criteria were children 4 years and older with a burn injury, hospitalized for >4 days.

For children under 4, therapists used the CARe. This form has several subscales and uses a Likert scale for rating. This project focused on subscales 2, 3, and 5, which assess parent confidence with burn care, social concerns about their child’s injury or scars, and parental stress, respectively. Sessions targeted building parent confidence and parents were appropriately referred if they indicated stress and social concerns. The CARe was administered to parents/caregivers of children 0-3 years old with a burn injury who were hospitalized >4 days.

**Results:**

For the AM-PAC, data was collected on 17 patients who met inclusion criteria from September 2022-September 2023. 8 patients were female and 9 were male. The average affected total body surface area was 8.5%. The average scores for the AM-PAC Basic Mobility at evaluation was 18 and at discharge was 19.3. The average scores for the AM-PAC Daily Activity at evaluation was 16.6 and at discharge was 17.8. 9 children demonstrated improvement.

For the CARe, data was collected on 12 patients who met inclusion criteria from September 2022-September 2023. The average age was 1.67 years old. The average score on the Confidence subscale was 26/35 at eval and the average score at discharge was 33.3/35. 10 patients demonstrated improvement from evaluation to discharge. Parents who reported social concerns and stress were referred to the unit Social Worker.

**Conclusions:**

The AM-PAC and CARe are feasible and helpful tools to employ in acute burn rehabilitation for pediatric patients. They allow therapists working with these patients to evaluate and treat the functional needs of the child and caregivers.

**Applicability of Research to Practice:**

This QI project adds to the body of knowledge about pediatric burn rehabilitation. More research is needed to evaluate the clinical utility of the AM-PAC and CARe. The presenters will continue to collect data and update the results prior to presentation if accepted.